# Anaphase A: Disassembling Microtubules Move Chromosomes toward Spindle Poles

**DOI:** 10.3390/biology6010015

**Published:** 2017-02-17

**Authors:** Charles L. Asbury

**Affiliations:** Department of Physiology & Biophysics, University of Washington, Seattle, WA 98195, USA; casbury@uw.edu

**Keywords:** anaphase A, kinetochore, chromosome-to-pole motion, microtubule poleward flux, conformational wave, biased diffusion

## Abstract

The separation of sister chromatids during anaphase is the culmination of mitosis and one of the most strikingly beautiful examples of cellular movement. It consists of two distinct processes: Anaphase A, the movement of chromosomes toward spindle poles via shortening of the connecting fibers, and anaphase B, separation of the two poles from one another via spindle elongation. I focus here on anaphase A chromosome-to-pole movement. The chapter begins by summarizing classical observations of chromosome movements, which support the current understanding of anaphase mechanisms. Live cell fluorescence microscopy studies showed that poleward chromosome movement is associated with disassembly of the kinetochore-attached microtubule fibers that link chromosomes to poles. Microtubule-marking techniques established that kinetochore-fiber disassembly often occurs through loss of tubulin subunits from the kinetochore-attached plus ends. In addition, kinetochore-fiber disassembly in many cells occurs partly through ‘flux’, where the microtubules flow continuously toward the poles and tubulin subunits are lost from minus ends. Molecular mechanistic models for how load-bearing attachments are maintained to disassembling microtubule ends, and how the forces are generated to drive these disassembly-coupled movements, are discussed.

## 1. Introduction and Distinction between Anaphase “A” and “B”

In his classic 1961 volume on cell division, Daniel Mazia referred to anaphase as the act of chromosome movement that gives mitosis its meaning [1] (p. 95). The term, anaphase, was originally coined over 130 years ago [2]. By Mazia’s time it had come to refer—as it still does today—to the phase of mitosis when sister chromatids are moving apart from one another toward opposite sides of the cell. The onset of anaphase is one of the most abrupt events of mitosis, making it cytologically useful as a reference for the timing of other mitotic events. It is also one of the most strikingly beautiful examples of cellular movement.

**Figure 1:**
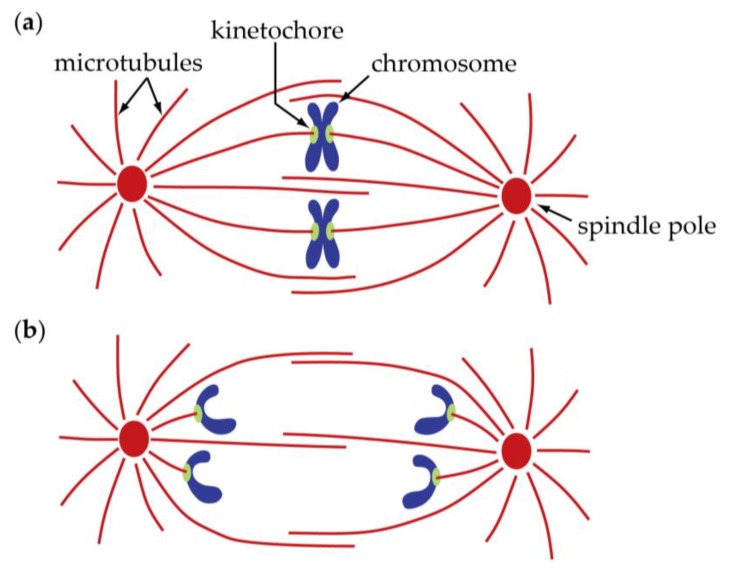
Schematic diagram of a spindle in metaphase (**a**) and anaphase (**b**). Only the chromosome-to-pole, “anaphase A” motion is depicted here; it is the focus of this chapter. Separation of the two spindle poles from one another via elongation of the spindle, “anaphase B”, is discussed in the subsequent chapter by Scholey et al. [3].

Anaphase consists of at least two distinct processes, traditionally referred to as “anaphase A” and “anaphase B”. Anaphase A is the movement of chromosomes toward the spindle poles via shortening of the connecting fibers; it is the focus of this chapter (Figure 1). Anaphase B, which is covered in the subsequent chapter by Scholey et al. [3], is the separation of the two poles from one another via elongation of the spindle. The distinction between anaphase A and B is more than a mere descriptive convenience. The two processes occur simultaneously in many cell types; but they are mechanistically distinct, a fact that has been appreciated since well before the underlying mechanisms were understood [4]. Anaphase A can be further divided into at least two mechanistically distinct sub-processes, as discussed below. 

This chapter begins with a description of chromosome movements during anaphase, which have been studied for over a century. Nevertheless, it is worthwhile to summarize the classical findings that support our current understanding and are sometimes taken for granted. Next is a description of microtubule dynamics within the spindle, another pillar of our modern view of anaphase. The remainder of the chapter is devoted to a discussion of force generation, which occurs also in earlier phases of mitosis but is most obvious during anaphase. Where and how are the forces that drive anaphase A generated? What roles are played by microtubule-based motor proteins and by the microtubules themselves? Evidence that the microtubules convert chemical energy into mechanical work is presented. Mechanistic concepts are emphasized, rather than specific molecules, with the hope that the discussion will be accessible and interesting, even for readers less familiar with mitosis.

## 2. Centromeres and Kinetochores Usually Lead Anaphase Movements While Chromosome Arms Follow

The idea that chromosomes are moved, during anaphase A and other phases as well, by forces exerted on them at kinetochores is so well established that the observations on which it rests are scarcely mentioned anymore. Condensed mitotic chromosomes are visible by brightfield microscopy, particularly when phase or differential interference contrast is used. Thus, as summarized in Chapter 1 of this volume [5], they have been observed for over a century. In certain cell types, the mitotic chromosomes are relatively long and their primary constrictions—their centromeres—are also discernable. Because these centromeric constrictions usually lead during mitotic chromosome movements (Figure 1), it is clear that they are major sites where force is transmitted to the chromosomes. Indeed, this is why they were given the name, kinetochores (“movement places”) [6]. Kinetochores in anaphase tend to move in straight paths toward the spindle poles, while the chromosome arms, following the kinetochores, swing and trace out more complex paths. Reflecting on these ‘rag-doll’ like movements, Mazia famously compared the role of chromosomes in mitosis to “that of a corpse at a funeral: they provide the reason for the proceedings but do not take an active part in them” [1] (p. 212).

**Figure 2:**
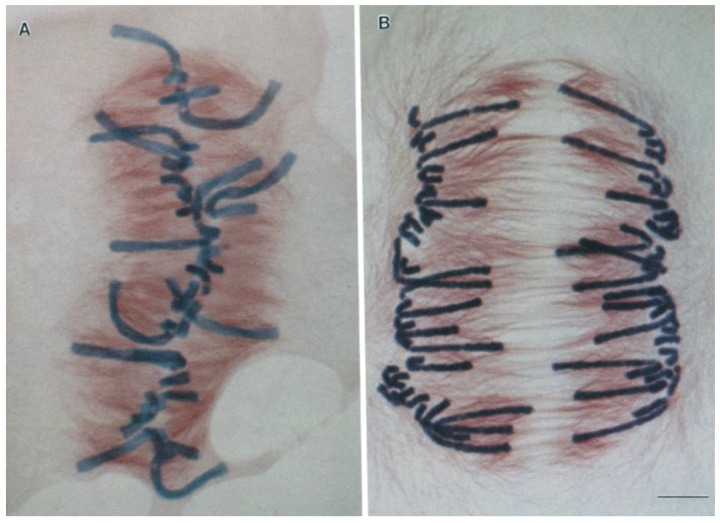
Light micrographs of metaphase (**a**) and late anaphase (**b**) plant endosperm (*Haemanthus*) spindles. During metaphase in these plant cells the chromosome arms are bent in the direction of the spindle poles. This behavior differs from what is seen in animal somatic cells, where chromosome arms are pushed continually away from spindle poles [11]. These *Haemanthus* images are reprinted from [9], and are displayed under the terms of a Creative Commons License (Attribution-Noncommerical-Share Alike 3.0 Unported license, as described at http://creativecommons.org/licenses/by-nc-sa/3.0/). Scale bar, 10 μm.

The general rule that kinetochores lead while chromosome arms follow applies in many cell types including vertebrates [7] and yeasts [8], but there are exceptions, such as in plant endosperm [9], and in crane-fly spermatocytes [10], where arms sometimes lead. These alternative cases remind us that forces are also exerted directly on chromosome arms, although the primary motive forces for anaphase are commonly exerted at kinetochores. The chromosome arms in certain well-studied mitotic cell types (e.g., Newt lung [7]) are pushed continually away from the spindle poles. These antipoleward forces have been dubbed the “polar winds” (or “polar ejection forces” [11]). They must be overcome by the kinetochores to drive anaphase poleward movement, and they explain why the chromosome arms usually point away from the poles in these cells. For other cell types, in which the chromosome arms sometimes lead the motion, the polar winds can blow toward, rather than away from the spindle poles [9,10]. Plant endosperm is an interesting case where chromosome arms first experience poleward forces prior to metaphase and then later, after anaphase onset, the situation reverses and arms experience away-from-the-pole forces (Figure 2) [9]. In crane-fly spermatocytes, however, poleward forces are apparently exerted on chromosome arms even during anaphase, providing an additional force that assists rather than opposes the kinetochores [10].

## 3. Poleward Movement during Anaphase A Is Mostly but Not Entirely Unidirectional

**Figure 3:**
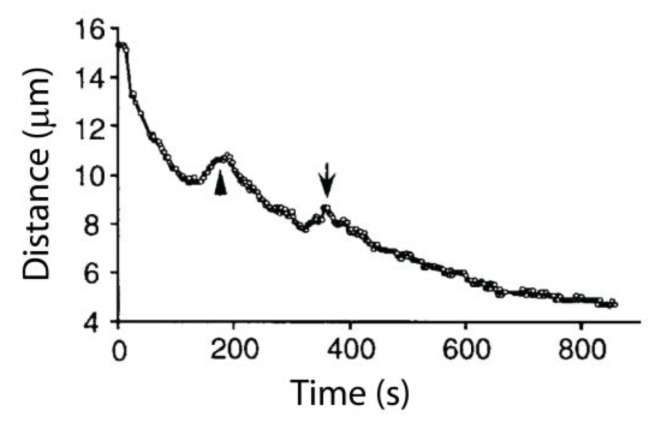
Example of kinetochore directional instability during anaphase A in a newt lung cell. Anaphase A chromosome-to-pole movement of the kinetochore is interrupted by transient reversals in directionality. This graph is reprinted from [12], and is displayed under the terms of a Creative Commons License (Attribution-Noncommerical-Share Alike 3.0 Unported license, as described at http://creativecommons.org/licenses/by-nc-sa/3.0/).

The poleward movement of kinetochores in anaphase is mostly unidirectional, but not always. Reversals in direction, similar to the oscillations seen earlier in prometaphase and metaphase, can continue in anaphase, but a poleward bias is generally maintained [12] (Figure 3). This bi-directional, back-and-forth movement has been named ‘directional instability’. It bears a striking resemblance to the intrinsic ‘dynamic instability’ of microtubule filaments, which stochastically switch between periods of shortening and growth [13,14], and suggests an intimate coupling between chromosome movements and microtubule dynamics, as discussed below. Although anaphase begins abruptly, anaphase chromosome movements are not perfectly synchronous. A kinetochore moving poleward in anaphase can reverse direction, transiently moving anti-poleward while its peers continue their poleward march. Neighboring chromosomes within a cell can also move closely past one another in opposite directions, e.g., when anaphase occurs prematurely, prior to formation of a proper metaphase plate (e.g., see [1] (p. 288) and [15]). A chromosome can also become erroneously attached to the spindle, with one of its kinetochores attached simultaneously to microtubules emanating from both poles. These “merotelically” attached chromosomes lag behind their properly (“amphitelically”) attached peers during anaphase [16]. Together these observations demonstrate that kinetochores are moved individually, rather than as a group. (Likewise, the mitotic error correction machinery acts at the individual kinetochore level, as described in the chapter in this volume by Grishchuk and Lampson [17].) 

Perhaps the most direct evidence supporting the primacy of kinetochores for moving chromosomes comes from UV ablation studies, which began as early as the 1950s [18]. If the kinetochores of a single chromosome are damaged by UV irradiation, the remaining chromosome arms drift rather than following their un-irradiated peers [7,18,19]. In contrast, a chromosome whose arm has been ablated follows the normal patterns of movement.

## 4. Poleward Chromosome Movement Is Coupled to Shortening of the Connecting Microtubules

Modern theories about chromosome movement began to emerge with the structural understanding of spindle architecture afforded by electron microscopy. Several distinct categories of microtubule filaments exist, with well-defined polarities (as discussed thoroughly in Chapter 1 of this volume [5]). The most important for anaphase A are the kinetochore-attached microtubules, which have one end, their fast-growing ‘plus’ end, located at a kinetochore, while their ‘minus’ ends project poleward. In medium-sized and larger spindles, many microtubules terminate together at each kinetochore and these are bundled together to form a kinetochore fiber. Some but not necessarily all the microtubules in a kinetochore fiber extend all the way to a spindle pole [20]. In the tiny spindles of budding yeast, the situation is simpler, with just one microtubule linking each kinetochore to a pole [21].

**Figure 4:**
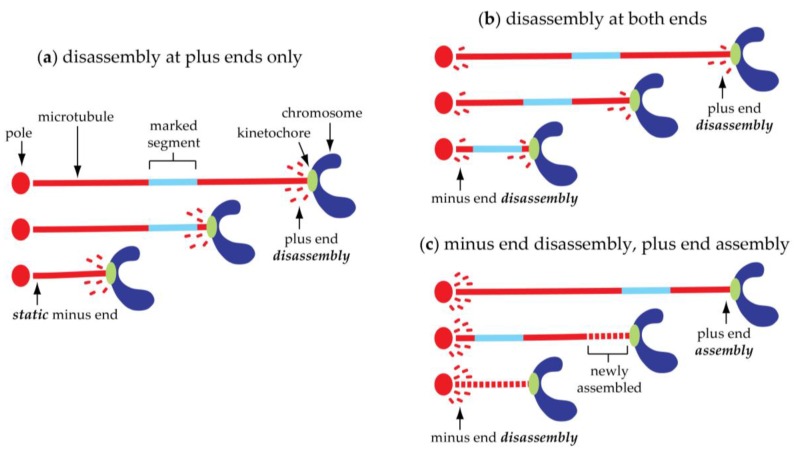
Chromosome-to-pole motion during anaphase A is coupled to microtubule disassembly. (**a**) Simple mechanism with disassembly occurring only at microtubule plus ends, as seen in yeasts, where minus end attachments to the poles are static and no flux occurs [26,27,28,29]. (**b**) Dual mechanism, as in cultured mitotic human cells, where chromosome-to-pole motion is a superposition of a kinetochore’s movement relative to the microtubules, which is coupled to plus end disassembly, and the microtubules’ flux relative to the poles, which is coupled to minus end disassembly [30]. (**c**) Mechanism observed for autosomal half-bivalents in meiotic crane-fly spermatocytes, with disassembly at minus ends and assembly at plus ends [10,31]. Switching between mechanism (b) and mechanism (c) has been directly observed in *Xenopus* egg extract spindles [32].

Advances in tubulin biochemistry and live-cell fluorescence microscopy have provided a fascinating view of the dynamics of microtubules in living spindles [22,23,24,25]. Time-lapse movies of large mammalian cells with fluorescent-tags on their kinetochores and their microtubules show that movement of a kinetochore is coupled to growth or shortening of the microtubule fibers to which it is attached [25]. During anaphase A, kinetochore-associated fibers shorten, without becoming noticeably thicker. This shortening of kinetochore fibers seems to draw the chromosomes poleward. In many cell types, microtubule-marking techniques (fluorescence photobleaching, photoactivation, and speckle microscopy) have shown that kinetochore fiber shortening occurs partly via loss of tubulin subunits from the kinetochore-attached plus ends (Figure 4a,b). How a kinetochore can maintain a persistent, load-bearing attachment to a microtubule tip that is disassembling under its grip is only poorly understood. Some models are discussed below.

## 5. Kinetochore-Attached Microtubules Can ‘Flux’ Continuously toward the Poles

Microtubule-marking techniques have also revealed that kinetochore-attached microtubules in many spindles flow steadily toward the poles (Figure 4b,c). This poleward microtubule ‘flux’ is coupled to minus end disassembly at or near the poles [23,31,32,33,34,35,36,37]. Anaphase A in these cells is therefore a superposition of a kinetochore’s movement relative to the microtubules and the microtubules’ flux relative to the poles. The contribution of flux to poleward kinetochore movement varies widely depending on cell type (Table 1). In mitotic human cells, for example, flux accounts for about a third of anaphase A chromosome-to-pole movement, the remaining two-thirds of which is due to plus end disassembly [30]. In budding or fission yeast, there is apparently no flux, so anaphase A in these cells is probably explained entirely by plus end disassembly [26,27,28,29]. In contrast, flux appears to be solely responsible for anaphase A in plant (tobacco) cells [37] and in meiotic crane-fly spermatocytes [10,31]. In the crane-fly spermatocytes, kinetochore-attached microtubule plus ends assemble, rather than disassembling during anaphase A. The bottom line is that microtubule fibers linking kinetochores to poles can disassemble from either end, or from both ends. The questions about how load-bearing attachments are maintained and how the speeds of movement are coordinated with rates of filament disassembly apply to both ends of the microtubules. 

## 6. Anaphase in Some Cell Types Does Not Conform to the Canonical View

A modern student of mitosis reading the classical literature cannot help but notice how many more types of cells were being examined. The advent of genetic and molecular approaches enabled a terrific array of tools that could not previously have been imagined. But these state-of-the-art tools have been aimed at a much more limited set of model cell types. And even within this limited set, there are examples that do not conform to the canonical view. Anaphase chromosome separation in the acentrosomal meiosis I spindles of *C. elegans* oocytes is apparently independent of kinetochores [54]. Instead, the chromosomes seem to be pushed from behind by microtubules growing and/or sliding out from the equator. The univalent X Y sex chromosomes in meiosis I crane-fly spindles move toward one pole while retaining microtubule fiber attachments to both poles [48]. The fiber on the trailing side elongates, while the leading fiber shortens. Probably more cases that do not fit the ‘normal’ picture will emerge as more transcriptomes and genomes are sequenced, and as new genome-editing technologies, such as CRISPR [55], facilitate live imaging of fluorescent-marked spindles in less-studied cell types

## 7. Kinetochores Can Either Be Actively Pulling Poleward or Passively Slipping Anti-Poleward

For a true, mechanistic understanding of anaphase, it is not enough simply to describe the motions of the kinetochores, the microtubules, and the poles relative to one another. We need to understand where and how the motive forces are generated. Biophysically, ‘force generation’ (or ‘force production’) refers to the *active* processes by which chemical energy, usually in the form of nucleotide triphosphates, is converted into mechanical work, defined as force acting through a distance. The forces that draw kinetochores toward spindle poles must be generated somewhere within the kinetochores themselves, within the poles, or within the material connecting them.

**Figure 5:**
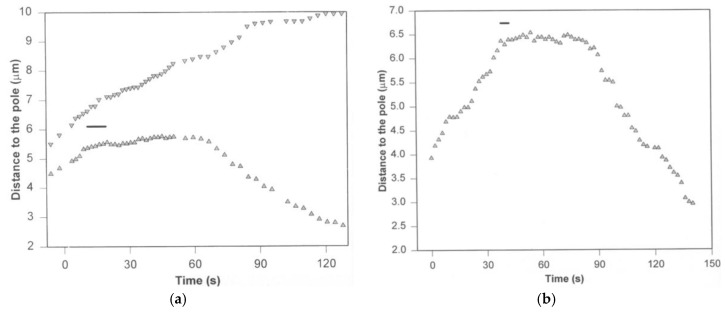
Kinetochores can adopt two distinct states, an active state that generates pole-directed pulling force, and a ‘neutral’ state that remains stationary or passively slips anti-poleward in response to external forces. (**a**) Motions of sister kinetochore regions in a metaphase PtK1 cell before, during (*horizontal bar*) and after micro-surgically separating the sisters. (**b**) Motion of a trailing kinetochore before, during (*horizontal bar*), and after selectively destroying its poleward moving sister kinetochore. In both cases the trailing kinetochore abruptly stops once it is micro-surgically freed from its sister. Then, after a ~20 s delay, it reverses its original directionality and begins to move poleward. These graphs are reprinted from [57], and are displayed under the terms of a Creative Commons License (Attribution-Noncommerical-Share Alike 3.0 Unported license, as described at http://creativecommons.org/licenses/by-nc-sa/3.0/).

The coupling of kinetochore movement to microtubule plus end disassembly strongly suggests that the kinetochore-microtubule interface is a site where force is actively generated. Compared to the early ablation studies that used UV-lamps [18], newer laser-equipped microscopes have enabled faster and more finely targeted ablations, making it possible in certain large cells (e.g., newt lung or PtK cells) to micro-surgically sever the centromeric chromatin connecting two sister kinetochores [56], or to selectively destroy one sister of a pair [57]. If a kinetochore moving poleward during metaphase is micro-surgically freed from its sister, it continues moving poleward (Figure 5a). However, if a kinetochore moving anti-poleward is freed, then it abruptly stops (Figure 5a,b), suggesting that its anti-poleward motion prior to the severing operation was a passive response to externally generated pulling forces (e.g., to forces generated by its poleward-moving sister) [57]. These observations, together with the highly coordinated oscillations of sisters in unperturbed cells [12], suggest that the force-producing machinery at a kinetochore can adopt two distinct states, an active state in which it generates pole-directed pulling force, and a ‘neutral’ state in which it remains stationary or passively slips anti-poleward in response to external forces. Such two-state behavior, with active minus end-directed pulling and passive plus end-directed slippage, is also observed when purified kinetochores are attached in vitro to dynamic microtubule tips ([58]; discussed further below). The behavior has implications for how a kinetochore’s force-generating machinery might operate, both before and after the metaphase-anaphase transition.

## 8. Anaphase Spindle Generates More Force than Needed for Anaphase Chromosome Movement

It might seem natural to assume that the spindle forces normally generated during anaphase, when the chromosomes are undergoing their most obvious movements, are higher than during other phases of mitosis. As Mazia [1] (p. 142) noted, “human laziness leads us to associate movement with hard work”. In anaphase, this assumption turns out to be false. However, the anaphase spindle is also capable of producing far more force than is normally necessary. 

Classic microneedle experiments, performed almost four decades ago, still provide some of the best and most direct measurements of spindle forces in anaphase. Nicklas used extremely thin, calibrated glass needles to tug on individual chromosomes in meiotic grasshopper spermatocytes and to ask how much opposing force was required to completely halt their chromosome-to-pole motion. The stall force he measured was surprisingly high, 700 pN [59]. This value represents the apparent limit of force production by the anaphase spindle in these cells—i.e., the maximum poleward force that the spindle can exert on a chromosome, presumably through its kinetochore(s). Nicklas assumed this load was shared by a subset of 15 kinetochore-attached microtubules that extended all the way to spindle pole (out of a total of ~40 kinetochore-attached microtubules), leading to an often-cited estimate of 50 pN per microtubule [59]. This might be an overestimate, with the true value falling closer to 12 pN per microtubule, given the recent work suggesting that all kinetochore-attached microtubules, even those that do not extend all the way to a pole, are anchored within the spindle [60,61]. But in either case the forces during a normal, unperturbed anaphase are probably much, much lower still. Viscous drag calculations suggest that chromosome-to-pole movement is normally driven by forces of only 0.1 pN [62]. Elastic bending of chromosomes likewise suggests only 0.7 pN [63]. Thus, the anaphase spindle can apparently exert a maximum poleward force (700 pN) that exceeds the normal anaphase force by as much as 1000- or even 7000-fold.

## 9. Why Is the Anaphase Spindle ‘Over-Engineered’ to Produce Forces so Much Higher than Needed?

What could be the evolutionary advantage of such an exceedingly high force-generating capacity? High capacity for force production might be advantageous during anaphase for disentangling chromosomes that remain inappropriately intertwined, perhaps helping to promote the decatenation activity of topoisomerases. High force-generating capacity might also be important during earlier stages of mitosis, before anaphase. During prometaphase, force at kinetochores provides a regulatory cue that promotes the selective stabilization of properly bioriented chromosome-spindle attachments. (See [64,65,66] and the chapter in this volume by Grishchuk and Lampson [17].) Kinetochore force might also be important for silencing the ‘wait’ signals generated by the spindle assembly checkpoint, which control entry into anaphase (as discussed in the chapter in this volume by Joglekar [67]). Bioriented kinetochores congressing to the spindle equator in prometaphase spermatocytes support intermediate levels of force, around 50 pN [68], which is much higher than the feeble forces normally seen in anaphase, <1 pN, but still less than the maximal value of 700 pN. Thus, the spindle might have evolved to pull forcefully against kinetochores *prior* to anaphase, to ensure that when anaphase does occur, the chromosomes will segregate correctly. In other words, the spindle’s capacity for producing very high forces during anaphase might be a byproduct of evolutionary pressure for high forces during earlier mitotic stages. Regardless of its evolutionary significance, the high force-generating capacity of the anaphase spindle has implications for the underlying mechanism of force production.

## 10. New Techniques Are Providing Force Estimates from a Wider Variety of Cell Types

Nicklas’ microneedle measurements were truly ground-breaking and their relevance to current mitosis research persists even four decades later. However, it should be noted that their generality is uncertain. Grasshopper spermatocytes are especially amenable to chromosome micromanipulation, probably because they lack a robust cortical layer of cytoskeletal filaments and thus their outer plasma membrane can be severely indented by a microneedle without being punctured or torn. (The needles do not puncture the membrane during successful experiments—accidental punctures cause cytoplasmic leakage and rapid cell death.) New techniques are needed for measuring kinetochore forces in other types of cells that are not amenable to micromanipulation.

Fluorescence-based approaches have recently shown great promise. By tracking the positional fluctuations of fluorescent centromeric probes, kinetochore forces during metaphase in budding yeast have recently been estimated at 4 to 6 pN [69]. This estimate agrees well with Nicklas’ prometaphase measurement of 50 pN, considering that the load on a grasshopper kinetochore is probably shared by numerous attached microtubules: Nicklas estimated 7 kinetochore-attached microtubules during prometaphase, each bearing 7 pN of load [68], whereas each kinetochore in budding yeast attaches just a single microtubule [21], bearing 4 to 6 pN. Calibrated fluorescence force-sensors inserted into the *Drosophila* kinetochore suggest somewhat higher loads during metaphase in this organism, 130 to 680 pN per kinetochore, or 12 to 62 pN per microtubule (assuming the load is shared by 11 microtubules) [70]. Thus, the forces sustained by kinetochore-microtubule junctions during normal prometaphase and metaphase might vary between 4 and ~60 pN, depending on the organism. How these pre-anaphase forces measured in yeast and *Drosophila* compare with the maximum force-generating capacity of their spindles is unknown, however, because the maximal force has only been measured in grasshopper spermatocytes.

Another potential approach for measuring kinetochore forces in living cells is to apply laser trapping. Calibrated laser traps have been used extensively for measuring forces produced by purified myosin, kinesin and dynein motors in vitro [71] and, more recently, to study isolated kinetochores and kinetochore subcomplexes coupled to microtubule tips in vitro (as discussed below). In a limited number of cases, laser traps have also been applied in living cells, to measure forces generated in vivo during the transport of small (and generally spherical) intracellular cargoes by kinesin and dynein motors in non-mitotic cells [72,73,74,75]. Because the standard methods for trap calibration cannot be applied in vivo, these studies have relied on external calibrations, performed after isolation of the trapped organelles (e.g., lipid droplets) from the cells [72,73,75], or they have used enhanced calibration methods that account for the viscoelastic behavior of cytoplasmic fluid [74,76]. Trap-induced photodamage, which is easily avoided in vitro by removal of dissolved oxygen [77], becomes a major concern whenever laser traps are applied in cells growing under aerobic conditions [78]. A recent study applying laser traps in meiotic spermatocytes from crane-fly and *Mesostoma* flatworms [79] suggests that the forces required to stall chromosome-to-pole movements in these cells might be ~100-fold lower than the 700 pN measured previously in grasshopper spermatocytes [59]. However, neither standard, nor enhanced trap calibration methods were used, and the chromosome movements were attenuated even after the laser trap was turned off, suggesting permanent photodamage rather than force-induced stalling.

## 11. Tip-Coupling: One of the Most Conserved Features of Mitosis and One of the Most Puzzling

The poleward movement of chromosomes coupled to shortening of microtubule plus ends is one of the most conserved features of mitosis. It is also one of the most puzzling. How is it possible for a kinetochore (or a spindle pole) to maintain a persistent and load-bearing grip on the end of a microtubule that is rapidly disassembling? Any proposed mechanism for anaphase A must explain this ‘tip-coupling’. A general mechanism should also be capable of explaining the other observations discussed above, such as the possibility for transient reversals in kinetochore directionality, the switching between active poleward and passive anti-poleward states, and the levels of force at kinetochores.

## 12. Conventional Motors Are Found at Kinetochores but Might Not Be the Primary Basis for Tip-Coupling

**Figure 6:**
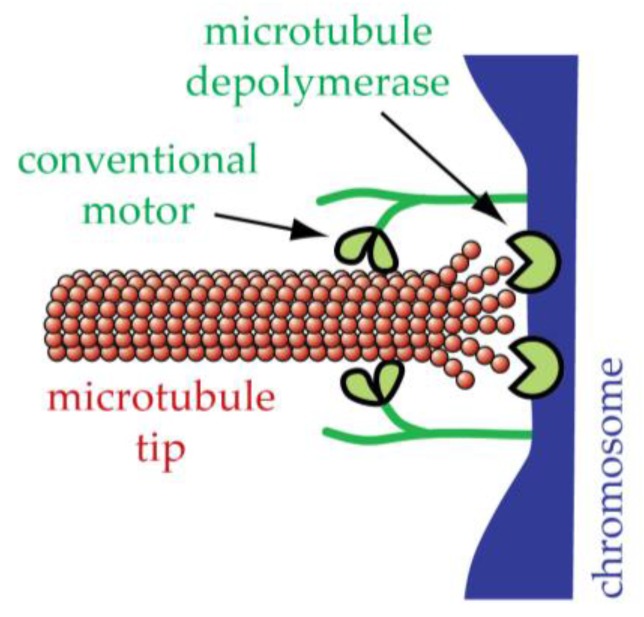
Model for kinetochore-microtubule tip-coupling based on conventional motor proteins and microtubule-regulators. Conventional ATP-powered, minus end-directed motor enzymes anchored at the kinetochore could reach around the tip of the microtubule, moving along the sides of the filament and thereby dragging the chromosome poleward (leftward in the diagram). The activities of additional microtubule depolymerases or severing enzymes, somehow coordinated with the conventional motor activity, could explain how poleward chromosome movement is coupled to plus end-disassembly.

Cytoplasmic dynein and kinesin-family motors were among the earliest molecules found to localize to centromeres [80,81,82] (closely following the seminal identification of CENP-A, -B, and -C [83]). Because ATP-powered motor enzymes are by themselves capable of moving along the sides of microtubule filaments, it is easy to imagine that they might represent the molecular basis for active force production at kinetochores. Minus end-directed motors anchored to a kinetochore could reach around the microtubule tip, moving along the sides of the filament and thereby dragging the chromosome poleward (Figure 6). Additional microtubule-modifying enzymes (microtubule depolymerases or severing enzymes) could explain how the motor-driven movement is coupled to plus end disassembly. Somehow the activities of these microtubule disassemblers would need to be coordinated with the motor enzymes.

There is good evidence that kinetochore-associated dynein contributes to anaphase A in certain cell types. Null mutations in the genes for zw10 or rod, components of the RZZ complex that links dynein to kinetochores, cause dramatic slowing of anaphase A chromosome-to-pole movement in *Drosophila* spermatocytes [84]. Acute inhibition of dynein by microinjection of excess p 50 ‘dynamitin’ (a component of the dynein-activating complex, dynactin) or of anti-dynein antibodies similarly slows anaphase A chromosome-to-pole speeds by ~75% in *Drosophila* embryos [85]. However, microinjenction-based inhibition of dynein in mammalian (PtK1) cells causes a much less-dramatic, ~33% slowing of anaphase A motion [86]. The chromosomes generally retain their attachments to dynamic microtubule plus ends [86], suggesting that kinetochore-associated dynein is dispensable for tip-coupling in these cells. Thus, while conventional motor proteins do play many vital roles during mitosis (especially for spindle assembly, prometaphase chromosome movements, and anaphase B, as discussed in the chapters in this volume by Kapoor [87], Goshima and Yamada [88], and Scholey et al. [3]), they do not seem to be the primary basis for tip-coupling. Dispensability of motor activity for tip-coupling in living cells is demonstrated most convincingly by studies of fission yeast, where poleward kinetochore movements coupled with microtubule disassembly can be directly observed even after all kinetochore-localized minus end-directed motors have been deleted [89]. Likewise, in budding yeast, disassembly-coupled kinetochore movements can continue in the absence of minus end-directed kinetochore motors [90]. More generally, deletion of various kinetochore-associated motors does not detach the kinetochores from the spindle [89,91,92,93,94]. These observations do not necessarily preclude a role for motors in tip-coupling, but they do argue against simple models in which tip-coupling is based primarily on a single type of conventional motor.

## 13. Kinetochores Also Contain Non-Motor Microtubule-Binding Elements

Our understanding of the biochemical composition and architecture of the kinetochore has grown immensely during the last decade (as discussed in the chapter in this volume by Musacchio and Desai [95]). The molecular details will not be repeated here, but the emerging view is that the kinetochore-microtubule interface includes an array of non-motor, microtubule binding proteins in addition to the conventional motors mentioned above. Foremost among these non-motor microtubule binders is the Ndc80 complex (Ndc80c), a fibrillar hetero-tetramer with one end that binds microtubules and another end that anchors stably into the core of the kinetochore [96,97,98,99,100]. Ndc80c localizes to the outer kinetochore layer, where microtubule tips are embedded, and its depletion causes widespread failure of kinetochore-microtubule attachment [101,102,103], suggesting a direct role in tip-coupling. Ndc80c is widely conserved. Its fibrillar structure contains hinge-points, enabling it to bend or fold [104,105,106]. Fluorescence measurements suggest that the relative abundance of Ndc80c (and other core subcomplexes) at individual kinetochores scales with the number of attached microtubules. Budding yeast kinetochores, which bind just one microtubule, are estimated to contain between 8 and 20 copies of Ndc80c [107,108]. Larger kinetochores that bind more microtubules have correspondingly more Ndc80c [109,110,111]. This scaling suggests modularity. The kinetochores of humans and other ‘higher’ eukaryotes might consist of large, parallel arrays of discrete microtubule-binding sites, each resembling a single budding yeast kinetochore [112].

Another microtubule-binding kinetochore element, specific to fungi, is the hetero-decameric Dam1 complex (Dam1c) [113,114,115]. Dam1c localizes to kinetochores in an Ndc80c-dependent manner and makes a major contribution to kinetochore-microtubule attachment in yeast [102,116]. Purified Dam1c spontaneously assembles into sixteen-membered, microtubule-encircling rings [117,118], which might function as sliding collars (as discussed below) [119,120]. The average number of Dam1 complexes per kinetochore is sufficient to form approximately one ring [107], or possibly two [108], per attached microtubule. Outside of fungi, the Ska complex has been proposed to provide a functionally similar activity [121,122], possibly via oligomerization, although it does not appear to form microtubule-encircling rings [122]. 

## 14. Toward an Integrated View of the Tip-Coupling Apparatus of the Kinetochore

The biochemical complexity of the kinetochore poses a major challenge for understanding how it functions. There are a variety of different microtubule-binding proteins likely to contribute, including the motor and non-motor proteins discussed above, and additional components as well. Unfortunately, our current understanding is too rudimentary to identify distinct roles for all of them. Current models for tip-coupling (and for other kinetochore functions as well, e.g., checkpoint signaling and error correction) emphasize the non-motor microtubule binders, especially Ndc80c and, in yeast, Dam1c. Kinetochore-anchored motor proteins are also very likely to be important. In principle, the kinetochore motors could participate in tip-coupling via their conventional ATP-powered walking along the sides of microtubules or, alternatively, they could participate in a manner independent of conventional walking motility [123,124,125,126,127]. That is, the kinetochore motors could function in tip-coupling essentially as fibrils that transiently bind and unbind from the microtubule, similarly to the non-motor microtubule binding fibril, Ndc80c. Another class of molecules likely to contribute are microtubule plus end-binders, such as those of the TOG (tumor overexpressed gene) family. TOG family proteins (Stu2 in budding yeast, XMAP215 in Xenopus, and chTOG in humans) localize to kinetochores [102,128,129,130,131,132,133,134] and contribute directly to tip-coupling in vitro [135,136]. The knockdown phenotypes for these plus end-binders, and for kinetochore motors, are often complex, suggesting roles in multiple different aspects of mitosis and making it difficult to assess specifically their roles in kinetochore tip-coupling in vivo.

An intriguing possibility is that the various microtubule-binders at kinetochores might interact with different structural features at the microtubule tip. For example, some might bind straight tubulins in the microtubule wall, while others might prefer curved protofilaments peeling out from the wall, and still others might even bind the longitudinal faces of tubulin dimers exposed uniquely at the extreme terminal subunits. More work is needed to test this idea. Especially useful would be better structural information about the relevant microtubule-binders, and more sophisticated biophysical methods for assessing the importance of specific microtubule contacts and specific tubulin conformations in kinetochore tip-coupling.

In the meantime, for the purpose of discussing potential biophysical mechanisms of tip-coupling, it seems sufficient at present to consider the kinetochore simply as a collection of flexible microtubule-binding fibrils, augmented in yeast (and possibly other organisms) by additional microtubule-binders that can potentially oligomerize into microtubule-encircling rings. This view is supported by the configuration of isolated yeast kinetochore particles seen in electron micrographs, which show 5 to 7 microtubule-binding fibrils connected to a central hub and sometimes associated with a microtubule-encircling ring [137]. It is also consistent with electron tomographic imaging of kinetochore-microtubule interfaces in vivo in multiple cell types [138,139].

## 15. Microtubules Could Be the Engines that Drive Poleward Chromosome Movement during Anaphase A

The tip-coupled movement of kinetochores implies force production at the kinetochore-microtubule interface. If conventional motor activity is dispensable, at least in some organisms, then how is energy transduced to drive this motility? Microtubules are likely to serve as the motors.

It is an old concept that anaphase A could be driven directly by the disassembly of spindle fibers. Inoue’s observations using polarization microscopy showed not only that the spindle was composed of birefringent fibers, but also that poleward chromosome movement could be induced by artificial dissolution of the birefringent material, using cold-treatment for example [140]. Enthusiasm for a fiber-driven mechanism might have temporarily waned after the discovery of motor proteins at kinetochores [140]. However, it apparently regained traction when improvements in the biochemical handling of tubulin enabled in vitro reconstitution of movement driven by microtubule disassembly [123,141], without ATP-powered motor activity [126] (reviewed in [140]). Further support has come from the discoveries that non-motor microtubule binders within the kinetochores are vital for kinetochore-spindle attachment in vivo, and that they can reconstitute tip-coupling in vitro. 

Microtubules are protein polymers composed of thousands of αβ-tubulins packed together in longitudinal rows, called ‘protofilaments’, that associate laterally to form a miniature tube [142]. In the presence of GTP, microtubules spontaneously self-assemble and they switch stochastically between periods of steady growth and rapid shortening, a behavior called ‘dynamic instability’ [13,14]. Dynamic instability is powered by GTP hydrolysis within αβ-tubulin. Growth occurs by addition of GTP-containing tubulins onto filament tips. Assembly triggers hydrolysis and phosphate release, so the body of a microtubule is composed primarily of GDP-tubulin, with ‘caps’ of GTP-tubulin at growing ends [143]. GDP-tubulin is intrinsically curved, but within the microtubule it is held straight—and therefore mechanically strained—by the bonds it forms with its lattice neighbors [144]. GTP-tubulin might be intrinsically straighter than GDP-tubulin [145], although recent work challenges this notion [146]. In any case, it is clear that some energy from GTP hydrolysis is retained within the GDP lattice [147,148], partly in the form of curvature-strain [143], and that this stored energy makes the microtubule unstable without protective end-caps. Severing the GTP-cap at a growing end triggers immediate disassembly [149]. During disassembly, the protofilaments first curl outward from the filament tip, releasing their curvature-strain, and then they break apart [144]. The energy released during tip disassembly can potentially be utilized to drive anaphase A chromosome-to-pole movement. 

## 16. Purified Kinetochores and Sub-Complexes Are Excellent Tip-Couplers

**Figure 7:**
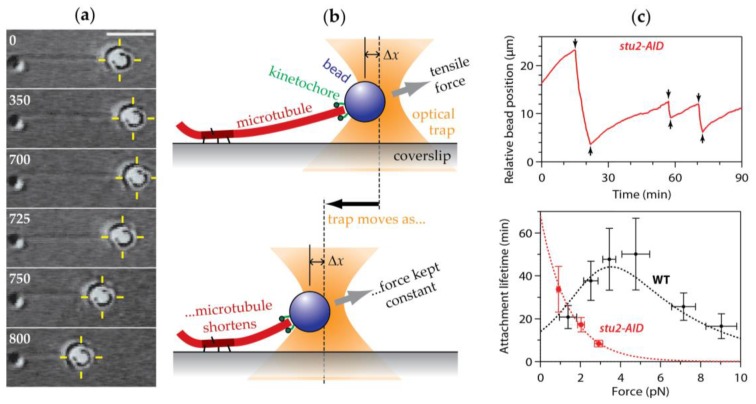
Laser trap assay for studying tip-coupling by purified kinetochore subcomplexes and native kinetochore particles. (**a**) Time-lapse images showing a bead decorated sparsely with native yeast kinetochore particles tracking with microtubule growth (0–700 s) and shortening (700–800 s). The laser trap (yellow crosshair) is moved automatically to keep a constant level of tension (here, ~1 pN) on the kinetochore as it moves with the microtubule tip. Scale bar, 4 μm. (**b**) Cartoon showing force clamp operation. The laser trap is servo-controlled to keep a fixed offset, Δ*x*, between the trap and the bead, thereby maintaining a constant tensile force. (**c**) *Upper plot:* Record of position versus time for a native kinetochore isolated from yeast cells depleted of the TOG-family protein, Stu2. Arrows indicate switching of the microtubule tip from growth to shortening (↓, ‘catastrophes’) and from shortening back to growth (↑, ‘rescues’). *Lower plot:* Mean attachment lifetime as a function of force for wild-type (WT, *black*) and Stu2-depleted (*stu2-AID*, *red*) kinetochore particles. Plots in (c) are adapted from [135], and are displayed with permission from Elsevier Publishing (http://www.sciencedirect.com/science/journal/00928674).

Direct evidence that energy can indeed be harnessed from disassembling microtubules comes from in vitro motility assays using purified kinetochore sub-complexes or isolated kinetochore particles to reconstitute disassembly-driven movement. With time-lapse fluorescence microscopy, oligomeric assemblies of recombinant fluorescent-tagged Ndc80c [150] or Dam1c [120,151,152] can be seen to track with shortening microtubule tips. Attaching the complexes to microbeads allows their manipulation with a laser trap and shows that they can track even when opposing force is applied continuously (Figure 7). The earliest laser trap assays of this kind used tip-couplers made from recombinant Dam1c or Ndc80c alone, which tracked against one or two piconewtons [119,150]. Coupling performance improved with the incorporation of additional microtubule-binding kinetochore elements [153,154], with the use of native kinetochore particles isolated from yeast [58], and with the use of flexible tethers for linking sub-complexes to beads [155]. Further improvements seem likely, especially as continued advancements in kinetochore biochemistry enable reconstitutions of ever more complete and stable kinetochore assemblies [156,157,158]. However, the performance achieved in laser trap tip-coupling assays already provides a reasonably good match to physiological conditions. Native budding yeast kinetochore particles remain attached to dynamic microtubule tips for 50 min on average while continuously supporting 5 pN of tension [58,135]. These statistics compare favorably with the total duration of budding yeast mitosis, which is typically <1 h, and with the estimated levels of kinetochore force in this organism, 4 to 6 pN [69]. Opposing forces up to 29 pN are needed to halt the disassembly-driven movement of tip-couplers made of recombinant Dam1c linked to beads via long tethers [155]. This stall force compares favorably with the estimated maximum poleward force produced per kinetochore-attached microtubule during anaphase A, which is between 12 and 50 pN (as discussed above) [68]. 

## 17. The Conformational Wave Model for Disassembly-Driven Movement

**Figure 8:**
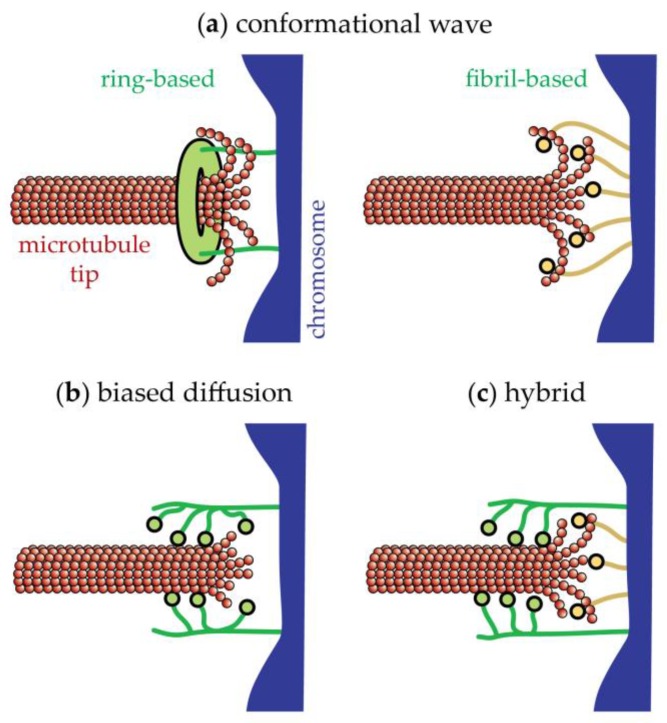
Models for tip-coupling without conventional motor activity. (**a**) Two versions of the conformational wave mechanism are shown, one (ring-based) in which elements of the kinetochore assemble into a microtubule encircling ring that is hooked by curling protofilaments, and another (fibril-based) where fibrillar kinetochore elements bind independently to the curling protofilaments. In either case, the curling action of the protofilaments exerts pulling force (directed leftward in the diagrams) on the chromosome. (**b**) In the biased diffusion mechanism, an array of kinetochore fibrils rapidly binds and unbinds the microtubule lattice at or near the tip. Thermal fluctuations of the chromosome that allow more fibrils to bind (leftward movements of the chromosome in the diagram) are favored by the energy of binding those elements. This biased thermal movement produces a thermodynamic pulling force. (**c**) A hybrid model is also shown, where force is produced by a combination of protofilament curling and biased thermal fluctuations. These diagrams are adapted from [159], and are displayed with permission from Elsevier Publishing (http://www.sciencedirect.com/science/journal/09628924).

Two classes of models are proposed to explain disassembly-driven movement of kinetochores, conformational wave and biased diffusion (Figure 8). According to the conformational wave model, the kinetochore literally surfs on the wave of curling protofilaments that propagates down a microtubule as it disassembles. To drive movement, the protofilaments are proposed to pull directly on the kinetochore as they curl outward from a disassembling tip [141]. Evidence supporting this model is compelling but not definitive [159]. Oligomeric Dam1c rings seem to be ideal structures for harnessing protofilament curls [117,118,160,161], and Dam1c does indeed make a major contribution to the stability and strength of kinetochore-microtubule coupling in vitro [58,162], acting as a processivity factor to enhance Ndc80c-based coupling [154,163]. The contribution of Dam1c to tip-coupling is highest when it is flexibly tethered [155] and when free Dam1c is also present in solution [152,162], presumably because these conditions facilitate oligomerization of Dam1c into a microtubule-encircling ring. A partial Dam1 sub-complex that is specifically deficient in oligomerization forms tip attachments that are far less stable than those formed by the full, wild-type complex [162]. However, direct evidence that the enhancements in tip-coupling afforded by Dam1c oligomers depend on curling protofilaments is lacking. Complete microtubule-encircling rings are not strictly necessary for Dam1c-based tip-coupling [151,152]. In principle, Dam1c rings could function by biased diffusion (as discussed in [159,164]).

Dam1c rings are not found outside fungi, but their absence does not necessarily rule out the conformational wave mechanism. Other molecules and structures could harness curling protofilaments. In humans and other eukaryotes, for example, the Ska complex might act as an attachment-stabilizer in a manner similar to Dam1c [121,122,165]. Ska complex does not appear to form oligomeric microtubule-encircling rings, but like Dam1c it can track with disassembling tips [166]. The Ska complex also dimerizes and might form lateral bridges between neighboring Ndc80 complexes [122]. Curling protofilaments might hook these lateral bridges. High-resolution electron tomograms show protofilaments curling outward from the tips of kinetochore-attached (and non-kinetochore) microtubules in mammalian (PtK1) spindles [138,167]. Sometimes fibrils can be discerned emanating from the kinetochores and connecting to the protofilament curls [138], suggesting the presence of a fibrillar protein with preferential affinity for curved protofilaments. Consistent with this possibility, the kinetochore protein Cenp-F contains an N-terminal microtubule-binding region that binds preferentially to ring- and curl-shaped tubulin oligomers (formed in the presence of dolastatin-10 and vinblastine, respectively) [168]. Beads decorated with N-terminal portions of Cenp-F can track with disassembling microtubule tips against forces of 3 pN [168], suggesting that its curl-binding activity could make a significant contribution to tip-coupling.

## 18. The Biased Diffusion Model for Disassembly-Driven Movement

Disassembly-driven kinetochore movement is also likely to depend partly on biased diffusion, a mechanism first proposed on purely theoretical grounds by Hill [169]. In this view the multiple microtubule-binding elements within a kinetochore form a diffusive attachment to the microtubule tip (Figure 8b). Thermal motions that bring more binding elements within reach of the tip are favored by the energy of binding those elements to the microtubule. Conversely, thermal motions away from the tip are disfavored because they reduce the number of binding elements that can reach the tip and thus they require some binding energy to be overcome. Hill showed theoretically that this bias is sufficient to allow persistent tracking with a disassembling microtubule tip, even against an external load.

Thermally driven diffusion along the microtubule lattice is a common property of many individual kinetochore proteins and subcomplexes. At the level of single molecules and small oligomers, Ndc80c [150], Dam1c [151], Ska complex [166], and Cenp-F [168] all bind and unbind quickly from microtubules and, while bound, diffuse rapidly over the lattice. When bound far from the microtubule tip and in the absence of external load their diffusive motion is random (the probability of movement in either direction is random) [150,151,166,168]. When they encounter a disassembling tip, a bias in their diffusion can be observed directly [150]. These behaviors fit strikingly well with the biased diffusion mechanism. Certain structural features of kinetochore subcomplexes also seem ideal for biased diffusion. Ndc80c [104], Dam1c [164], Ska complex [122], and Cenp-F [170] all appear to bind microtubules through flexible domains, which could allow some to bear load while others unbind and rebind in new locations, enabling a kinetochore to move or reorient on the microtubule without detaching. Diffusion along the microtubule lattice is negligibly slow for large assemblies of Dam1c [152] and for whole native kinetochore particles [58], but these observations do not rule out biased diffusion as a mechanism for tip-coupling by these assemblies. Large couplers that contain high numbers of microtubule-binders are not expected to diffuse detectably along the lattice, but they can nevertheless track robustly with a disassembling tip via pure biased diffusion [150,169]. Robust tip-tracking occurs in these cases, despite low mobility on the lattice, because the diffusional mobility increases as the tip begins to disassemble out from under the coupler. This in turn promotes lattice-directed movement and formation of new bonds, resulting in a steady state where the rate of new bond formation is balanced by the loss due to disassembly.

## 19. Movement Coupled to Tip Assembly

Reconstituted tip-couplers made from various combinations of kinetochore subcomplexes [119,154] and from native kinetochore particles [58,135,171,172] can also maintain persistent, tension-bearing attachments to *assembling* tips (e.g., see Figure 7). Their assembly-coupled movement in vitro is analogous to situations in vivo when kinetochores move anti-poleward in association with growing microtubule tips, such as during pre-anaphase chromosome oscillations, or during transient reversals of anaphase A chromosome-to-pole movement. The reconstituted couplers generally adopt a ‘neutral’ state, very much like that of kinetochores moving anti-poleward in vivo, requiring external tension to track with tip growth rather than being pushed autonomously by the growing tip. Affinity between the coupler and the microtubule creates a protein friction that resists movement along the filament [169]—an effect sometimes refered to as a ‘slip clutch’ [32]. Considering that curled protofilaments are much less prominent at assembling tips in vitro [144], and that the conformational wave mechanism is based on curled protofilaments, a purely conformational wave-based coupler would be expected to detach more quickly during assembly than during disassembly. But just the opposite is true: The reconstituted couplers usually detach far *less* quickly from assembling tips [58,135,171]. 

Based on electron tomographic studies of microtubule tips in cells, it has been suggested that protofilaments might curl out from *both* disassembling *and* assembling tips in vivo [138,139,173]. However, many of the kinetochore-attached plus ends examined in another electron tomographic study were apparently blunt, with straight protofilaments [167]. And in cells treated with nocodazole to promote tip disassembly, the same study found that kinetochore-attached microtubule ends were predominantly flared, with curling protofilaments [167], supporting the general view that curling protofilaments are restricted mainly to disassembling tips in vivo, as in vitro. Sheet-like extensions or blunt structures, not curls, have also been reported at assembling microtubule tips in mitotic and interphase cell extracts [174,175,176]. A purely conformational wave-based coupler should detach very quickly from these blunt microtubule ends. The biased diffusion mechanism has fewer structural constraints and could maintain a stable attachment independent of microtubule tip structure.

## 20. Mechanism of Poleward Flux Might Differ for Kinetochore-Attached Versus Non-Kinetochore Microtubules

Poleward microtubule flux contributes to anaphase A chromosome-to-pole motion in many organisms (Table 1). At a cellular level flux seems like a very close cousin to the movement of kinetochores relative to microtubule plus ends. Flux is coupled to disassembly of the pole-facing minus ends of spindle microtubules, just as kinetochore movement is coupled to plus end disassembly. Flux suggests force production at or near the depolymerizing minus ends, just as disassembly-coupled kinetochore movement suggests force production at plus ends. The speeds of both processes depend on some of the same types of microtubule regulatory molecules. Whether they share fundamentally similar mechanisms, however, is unclear. 

The molecular and biophysical basis for poleward flux of non-kinetochore microtubules is reasonably well understood, but the same cannot be said for the flux of kinetochore-attached microtubules. Some non-kinetochore microtubules emanating from opposite spindle poles interdigitate within the central spindle to form antiparallel bundles—the so-called ‘inter-polar microtubules’ [3]. These bundles are held together by a collection of microtubule cross-linking proteins, including kinesin-5s, which are bipolar (tetrameric), processive, plus end-directed motors [3]. Individual purified kinesin-5 molecules can bind two antiparallel microtubules in vitro and simultaneously walk toward both plus ends, thereby driving outward protrusion of the minus ends [177]. Thus kinesin-5s appear to be perfectly suited for pushing inter-polar microtubules outward and driving their flux. But kinetochore-attached microtubules generally have parallel polarity [178], not antiparallel, and therefore their flux cannot be explained by a direct, antiparallel sliding action. Kinetochore-attached microtubules can associate laterally with non-kinetochore microtubules [20,60], and it has been suggested that perhaps the flux of kinetochore microtubules is driven indirectly, by the flux of their laterally associated neighbors (e.g., see [179]).

Alternatively, the mechanisms driving kinetochore-microtubule flux might differ from those driving non-kinetochore microtubule flux. Pharmacological inhibition of kinesin-5 dramatically slows flux in *Xenopus* extract spindles, in which a majority of microtubules are non-kinetochore-associated [180,181]. But in cultured mammalian (PtK1) cells, where a large proportion of microtubules are kinetochore-attached, kinesin-5 inhibition has only a minor effect on flux rates [179]. Furthermore, flux continues even when the spindles are monopolar, and therefore lacking antiparallel microtubules [179], indicating that neither kinesin-5 nor antiparallel microtubules are required for flux in these cells. Likewise, kinetochore-associated microtubule fibers that are mechanically detached and isolated from spindles in grasshopper spermatocytes flux in the apparent absence of antiparallel neighboring microtubules [182]. Thus, it seems that flux of kinetochore-attached microtubules can be driven by another mechanism, independent of the kinesin-5-dependent sliding of neighboring, antiparallel (inter-polar) microtubules.

## 21. Potential Biophysical Mechanisms for Kinetochore-Microtubule Flux

Flux generally depends on the activity of microtubule destabilizing enzymes that concentrate at spindle poles. Enzymes of the kinesin-13 family are ATP-powered depolymerases that catalyze the disassembly of microtubules by removal of tubulin subunits from their ends [183,184,185]. Kinesin-13s concentrate at poles in various spindle types, such as those in mitotic *Drosophila* cells [36], human cells [186,187], and frog cell extracts [188]. Depletion of the pole-localized *Drosophila* kinesin-13, KLP10A, specifically slows microtubule flux in this organism, and concomitantly reduces the speed of anaphase A chromosome-to-pole motion [36,45,46]. Similarly, the flux component of anaphase A in mitotic human cells is slowed by co-depletion of a pole-localized and a centromere-associated kinesin-13, Kif2a and MCAK, respectively [30]. The AAA-family microtubule-severing enzymes, spastin and fidgetin, are also implicated in poleward microtubule flux in *Drosophila* [44,189] and human cells [52]. Their severing activity might be important for creating free microtubule minus ends (i.e., not capped by γ-tubulin rings) and thereby facilitating the catalysis of minus end disassembly by kinesin-13s. Collectively these observations indicate that microtubule destabilization activity at poles governs the rate of flux. But a governor is not necessarily a motor. The microtubule-destabilizers might or might not be directly involved in maintenance of load-bearing attachments between microtubules and spindle poles, or in the production of forces that drive flux. In some cells, microtubule depolymerizers also govern the speed of disassembly-coupled kinetochore movement [36,45,53], yet they are not usually considered to be the primary force-producers. 

What then is the flux engine? One could envision a conformational wave- or biased diffusion-based tip-coupling that directly harnesses the energy released from minus end disassembly, analogous to the mechanisms discussed above for kinetochore motility. Whether spindle poles carry microtubule-binding elements with the properties necessary to support such tip-coupling is uncertain, but some evidence suggests so: The pole-localized *Drosophila* kinesin-13, KLP10A has been found to oligomerize into microtubule encircling rings [190,191], reminiscent of the Dam1c rings implicated in kinetochore tip-coupling. Other kinesin-13s, such as *Drosophila* KLP59C and human MCAK, which localize primarily near centromeres [36,192], can likewise form oligomeric rings around microtubules [190], and MCAK can function as a tip-coupler in vitro [193]. Together these observations suggest that kinesin-13s might function not only as depolymerizers but also as tip-couplers at spindle poles, and possibly at kinetochores as well.

Minus end-directed motors, particularly dynein, might also be involved in driving poleward microtubule flux. Dynein helps focus microtubules into poles in a variety of cell types. Pole focusing by motors is perhaps best understood in mitotic *Xenopus* egg extracts, where the minus end-directed movement of dynein oligomers can bring minus ends together to form polarized microtubule asters independently of centrosomal nucleation [194,195]. Dynein is also implicated in pole-focusing in *Drosophila* (S2 [196]) and mammalian cells (monkey kidney CV-1 [197,198]; rat-kangaroo PtK2 [60]; human RPE1 [61]). Pole-focusing by dynein probably requires oligomerization [199,200] via interaction with scaffolding proteins such as NuMA [195,198], and its importance for assembly and maintenance of bipolar spindles has been studied extensively.

**Figure 9:**
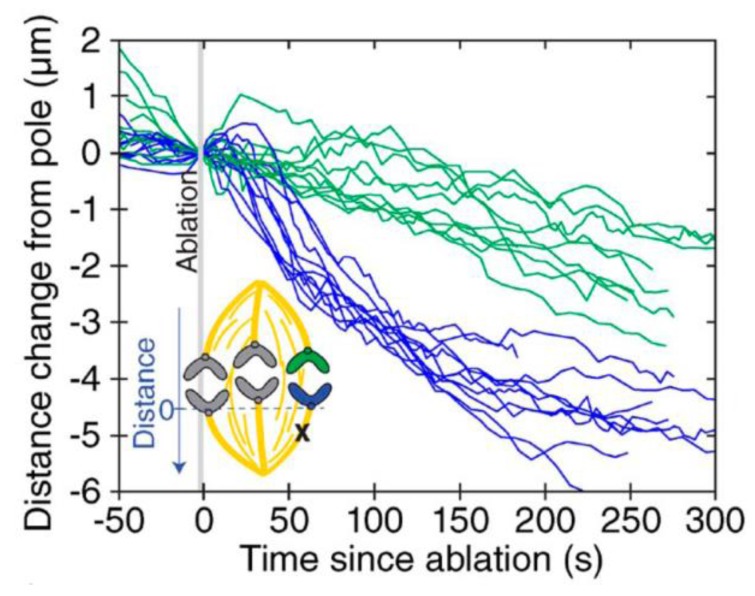
Change in distance from chromatids to poles before and after ablation of their kinetochore-associated microtubule fibers (k-fibers) during anaphase. Chromatids attached to ablated k-fibers (*blue traces*) are pulled toward poles faster than anaphase movement of their unmanipulated sisters (*green traces*) before resuming normal anaphase movement (at ~70 s). This graph is reprinted from [60], and is displayed under the terms of a Creative Commons License (Attribution-Noncommerical-Share Alike 3.0 Unported license, as described at http://creativecommons.org/licenses/by-nc-sa/3.0/).

**Figure 10:**
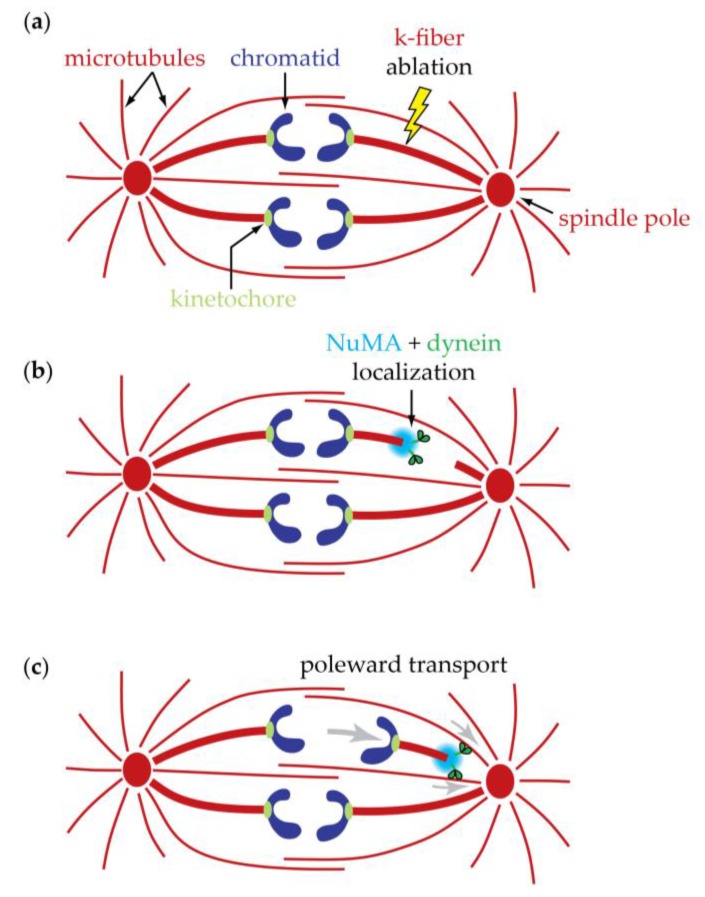
Spindle self-repair mechanism observed after micro-surgical ablation of kinetochore-associated microtubule fibers (k-fibers) in mammalian cells expressing fluorescent tubulin [60,61]. (**a**) Ablation of a k-fiber (*yellow lightning bolt*) during anaphase. (**b**) NuMA (*cyan*) and dynein/dynactin (*green*) rapidly localize to new microtubule minus ends on the k-fiber stub after ablation. (**c**) When the new minus end-localized dynein contacts neighboring microtubules, it walks processively along them, pulling the k-fiber stub as cargo and moving the attached chromosome. These diagrams are redrawn based on similar cartoons from [60], and are included here under the terms of a Creative Commons License (Attribution-Noncommerical-Share Alike 3.0 Unported license, as described at http://creativecommons.org/licenses/by-nc-sa/3.0/).

Recent work implicates dynein in poleward movement specifically of kinetochore-attached microtubules. Bundles of kinetochore-attached microtubules that do not extend all the way to the spindle pole are sometimes seen in normal spindles [61,196] and can also be created artificially by laser micro-surgery [60,61]. When a microtubule fiber attached to one kinetochore of a bioriented pair is micro-surgically severed during metaphase, the cut fiber stub and its attached kinetochore initially recoil toward the sister kinetochore on the uncut side, as the chromatin linking the two sisters relaxes. This relaxation is expected due to the sudden loss of tension. But within a few tens of seconds the fiber stub is suddenly jerked poleward [60,61]. If a fiber is severed in early anaphase, the behavior is similar: There is no obvious initial recoil, presumably because sister chromatin cohesion is absent in anaphase, but the fiber stub and kinetochore are suddenly jerked poleward (Figure 9) [61], just as they are in metaphase. The poleward-facing ends of the fiber stubs lead these rapid poleward movements, apparently by associating laterally with nearby, uncut microtubules. Fluorescence imaging reveals rapid recruitment of NuMA and dynein to the newly created minus ends. Presumably this dynein drives poleward movement of the minus ends along neighboring pole-anchored microtubules (Figure 10). This activity was seen in both pre-anaphase and anaphase cells, and it shows that the spindle is capable of remarkable acts of self-healing. Whether a similar mechanism could drive the steady flux of kinetochore-attached microtubules during anaphase is uncertain. The idea seems attractive, although flux in *Drosophila* S2 cells has been shown to be independent of dynein [201]. Questions also remain about how depolymerase activity is engaged when the motors and minus ends reach the pole.

## 22. Loss of Tension by Itself Might Be Sufficient to Trigger Anaphase Chromosome-to-Pole Movement

Having surveyed possible mechanisms underlying chromosome-to-pole motion during anaphase A, it is interesting to return briefly, at the end of the chapter, to the beginning of anaphase. Anaphase begins abruptly. Cohesion between sister chromatids is proteolytically removed, essentially simultaneously from all sister pairs. Mechanical tension on all the kinetochores is suddenly lost. Is this sudden loss of tension, by itself, sufficient to trigger the poleward motion of kinetochores? Or is the inherent activity of the anaphase machinery modulated by regulatory cues at the metaphase-to-anaphase transition?

Ever since Östergren, a compelling hypothesis has been that the same mechanisms might account for both the alignment of chromosomes at metaphase and also their poleward movement at anaphase (e.g., see [140,202,203]). Micro-surgical studies support this view. When a kinetochore moving anti-poleward during metaphase is stopped by ablation of its sister (as described above), it stops only transiently, for ~20 s, and then begins to move poleward—i.e., with reversed, anaphase-like directionality [57]. This transition to poleward movement is apparently caused by the loss of tension when a chromatid is cut free from its sister. The anaphase-like poleward movement might be triggered in this case because micro-surgically severing the sisters closely mimics the normal trigger of anaphase, enzymatic removal of sister chromatid cohesion. Both operations cause a sudden loss of tension across the sisters.

In vitro reconstitutions of tip-coupling show directly that regulatory cues are not needed to trigger disassembly-driven kinetochore movement. Tension applied through Dam1c-based tip-couplers [204] or through native yeast kinetochore particles [58,135] promotes net growth of the attached microtubule. Tension speeds tip assembly, slows disassembly, inhibits switches from growth to shortening (‘catastrophes’), and promotes the resumption of growth (‘rescues’) [58,135]. The effect of tension on catastrophe frequency is especially dramatic: At modest concentrations of free tubulin, the growth of a bare microtubule tip will typically persist for only a few minutes before a catastrophe occurs. Association of a relaxed kinetochore with the tip extends this uninterrupted growth time to ~8 min, but catastrophes are still relatively frequent. Applying a tension of 6 pN, however, can extend the uninterrupted growth time 13-fold, to over 100 min [58]. Thus, it is possible to experimentally induce a long period of assembly-coupled kinetochore movement by applying 6 pN of tension, and then to trigger disassembly-driven movement at will, simply by dropping the tension [205].

## 23. Phosphoregulatory Changes at the Metaphase-to-Anaphase Transition

While the simple loss of tension is sufficient to trigger an anaphase A-like switch in kinetochore directionality in vivo [57,140] and in vitro [58,135,205], it would be naïve to assume that the anaphase machinery is un-regulated during the true metaphase-to-anaphase transition in vivo. By now it is clear that multiple distinct mechanisms can underlie almost every aspect of mitosis. The same biochemical signaling cascade that brings about the sudden proteolytic destruction of sister cohesion also destroys cyclin B, thereby deactivating the cyclin-dependent kinase, CDK1, and causing a variety of global cellular changes associated with mitotic exit. Cyclin B and CDK1 are known to regulate microtubule dynamics (e.g., see [194,206]) and loss of cyclin B is proposed to stabilize inter-polar microtubules to promote anaphase B spindle elongation ([207]; as also discussed in the subsequent chapter on anaphase B [3]). If kinetochore-attached microtubules were similarly stabilized, the effect on anaphase A would be antagonistic, potentially slowing chromosome-to-pole movement by retarding disassembly at both plus and minus ends. However, evidence from budding yeast [206] and human tissue culture cells [208] indicates that the dephosphorylation associated with deactivation of CDK1 (or with activation of its antagonizing phosphatase, Cdc15) helps to promote, rather than antagonize anaphase A. In human cells, chemical inhibition of dephosphorylation converts the normally smooth chromosome-to-pole motion, with few reversals, into a much more oscillatory motion, with frequent reversals [208]. 

Another consequence of deactivating CDK1 is release of Aurora B kinase from centromeres (along with its co-members in the chromosomal passenger complex). Releasing Aurora B ensures that the sudden loss of kinetochore tension at anaphase onset does not activate the prometaphase error correction machinery, which would otherwise destabilize kinetochore-microtubule attachments. (Error correction is discussed in detail in the chapter by Grishchuk and Lampson [17].) This freeing of kinetochores from the influence of Aurora B should strengthen their attachments to spindle microtubules and, indeed, Nicklas noted in his early micromanipulation experiments that chromosomes became more difficult to detach as cells progressed from prometaphase into anaphase [209]. Freeing kinetochores from the influence of Aurora B might also affect the dynamics of kinetochore-attached microtubule plus ends: Aurora inhibitors stabilize kinetochore-attached microtubules in cells [210] and, conversely, phosphomimetic mutations at Aurora B target sites on Ndc80c and Dam1c destabilize kinetochore-attached plus ends in vitro [171,211]. Both observations implicate Aurora B in destabilization of kinetochore-attached plus ends. Thus, removal of Aurora B at anaphase onset should cause stabilization of the kinetochore-attached ends, which would be antagonistic toward anaphase A chromosome-to-pole movement. Perhaps the microtubule-stabilizing effects caused by loss of Aurora B are sufficiently counteracted by the destabilization due to loss of tension, or by other as-yet-unidentified regulatory events. Clearly more work is needed to understand how phosphoregulatory changes at anaphase onset regulate chromosome-to-pole motion. 

## 24. Conclusions

Anaphase is the dramatic finale of mitosis when, after careful preparations are finished, the actual business of segregating duplicated chromosomes takes place in a beautifully orchestrated manner. Kinetochores are the main sites where forces are exerted on the chromosomes. The interfaces between kinetochores and microtubule plus ends are primary sites where forces are produced to drive anaphase A chromosome-to-pole movement. The microtubules themselves are likely to act as non-conventional motors, converting chemical energy from GTP hydrolysis into mechanical strain, storing this strain energy temporarily in their lattices, and then releasing it during disassembly. The released energy is harnessed in part by non-motor, microtubule-binding kinetochore elements, perhaps via surfing on waves of curling protofilaments. Meanwhile, in many cell types the kinetochore-attached microtubules are also transported steadily poleward, by mechanisms that are not yet well understood. This poleward flux supplements kinetochore tip-surfing. Chromosome-to-pole motion is likely triggered at the metaphase-to-anaphase transition in part by the simple loss of tension that occurs when cohesion between sister chromatids is suddenly lost, but additional phosphoregulatory influences are also important.

## Figures and Tables

**Table 1:** Speeds of chromosome-to-pole anaphase A motion, and microtubule-to-pole flux motion, measured in various spindle/cell types.

Spindle/Cell Type	Chromosome-to-Pole Speed (μm/min)	Speed Measured in Anaphase A?	Microtubule-to-Pole Flux Speed (μm/min)	Technique for Flux Measurement	Flux Measured in Anaphase A?	Experimental Condition	Fraction of Anaphase A Speed Due to Flux (%)	Reference
Sand dollar embryos	1	yes	1.8	photobleaching	yes	control	180	[38]
Newt lung cells	1.7	yes	0.44	photoactivation	yes	early anaphase	26	[33]
	0.54	yes	0.18	photoactivation	yes	late anaphase	33	
Newt lung cells	0.2	yes	0.2	photoactivation	yes	10 uM taxol, late anaphase	100	[39]
Pig kidney (LLC-PK) and rat kangaroo (PtK1) cells	1.2	yes	0.2	photoactivation	yes	early anaphase	17	[34]
*Xenopus* (meiotic) extract spindles	2	yes	2	photoactivation	yes	control	100	[40]
	0.2	yes	0.2	photoactivation	yes	1.5 mM AMPPNP	100	
	2	yes	2	photoactivation	no (metaphase)	1 uM taxol	100	
*Xenopus* (meiotic) extract spindles	2.8	yes	1.6	speckle	yes	plus end depol.	57	[32]
	0.7	yes	1.6	speckle	yes	plus end polym.	229	
Budding yeast	1.3	yes	-	-	-	CEN dots (14 kb LacO array)	-	[8]
Budding yeast	-	-	0	photobleaching	no (anaphase B)	ipMTs (not kMTs)	-	[26]
Budding yeast	0.3	yes	-	-	-	CEN dots (2–11 kb LacO arrays)	-	[41]
Budding yeast	0.3	yes	-	-	-	CEN dots (2 kb LacO array)	-	[42]
Fission yeast	-	-	0	photobleaching	no (anaphase B)	ipMTs (not kMTs)	-	[29]
Fission yeast	-	-	0	speckle	no (anaphase B)	ipMTs (not kMTs)	-	[28]
Fission yeast	-	-	0	photobleaching	no (anaphase B)	ipMTs (not kMTs)	-	[27]
*Drosophila* embryos	3.6	yes	3.2	speckle	yes	control, 18 °C	89	[35]
*Drosophila* embryos	6.4	yes	1.9	speckle	yes	control	30	[43]
*Drosophila* embryos	5.6	yes	2.2	speckle	yes	control	39	[36]
	3.4	yes	3.4	speckle	yes	anti-KLP59C	100	
	3.2	yes	0	speckle	no (metaphase)	anti-KLP10A	0	
*Drosophila* (S2) cells	1.2	yes	0.6	photobleaching	yes	control	50	[44]
	0.6	yes	0.5	photobleaching	yes	katanin RNAi	83	
	0.8	yes	0.2	photobleaching	yes	spastin RNAi	25	
	0.7	yes	0.1	photobleaching	yes	fidgetin RNAi	14	
*Drosophila* (S2) cells	1.7	yes	0.9	photobleaching	yes	control	53	[45]
	0.7	yes	0.5	photobleaching	yes	KLP59D RNAi	71	
	1.7	yes	0.9	photobleaching	yes	KLP59C RNAi	53	
	0.8	yes	0.3	photobleaching	yes	KLP10A RNAi	38	
*Drosophila* (S2) cells	0.8	yes	0.4	speckle	yes	control	50	[46]
	0.7	yes	0.2	speckle	yes	CLASP & KLP10A RNAi	28	
*Drosophila* spermatocytes (meiosis)	-	-	0.6	photobleaching	no (metaphase)	metaphase	-	[47]
	1.7	yes	0.6	photobleaching	yes	disjoining	35	
	2.7	yes	1	photobleaching	yes	separated	37	
Crane-fly spermatocytes (meiosis)	0.5	yes	0.9	speckle	yes	autosomal half-bivalents	180	[31]
Crane-fly spermatocytes (meiosis)	1.3	yes	0.8	speckle	yes	sex univalent, bipolar link cut	62	[48]
Crane-fly spermatocytes (meiosis)	0.7	no (metaphase)	0.7	speckle	no (metaphase)	autosomal, cut K-fragment	100	[49]
Human (U20S) cells	1.5	yes	0.5	photoactivation	no (metaphase)	control	33	[30]
	1.2	yes	0	photoactivation	no (metaphase)	MCAK & Kif2a RNAi	0	
Human (U20S) cells	-	-	0.5	photoactivation	no (metaphase)	control	-	[50]
	-	-	0.3	photoactivation	no (metaphase)	CLASPs RNAi	-	
	-	-	0.3	photoactivation	no (metaphase)	Cenp-E RNAi	-	
Human (U20S) cells	-	-	0.6	photoconversion	no (metaphase)	control	-	[51]
	-	-	0.3	photoconversion	no (metaphase)	Kif4A RNAi	-	
Human (U20S) cells	0.44	yes	0.9	photoactivation	no (metaphase)	control	-	[52]
	0.3	yes	0.6	photoactivation	no (metaphase)	fidgetin siRNA	-	
Human (HeLa) cells	-	-	0.4	photoactivation	no (metaphase)	control	-	[53]
	-	-	0.2	photoactivation	no (metaphase)	ectopic MCAK at CEN	-	
Human (HeLa) cells	1.7	yes	-	-	-	control	-	[15]
	0.9	yes	-	-	-	Kif18A overexpress.	-	
	2.8	yes	-	-	-	Kif18A siRNA	-	
Tobacco (BY-2) cells	2.1	yes	2	photobleaching	yes	control	95	[37]
